# Bibliometric analysis of peer-reviewed literature on antimicrobial stewardship from 1990 to 2019

**DOI:** 10.1186/s12992-020-00651-7

**Published:** 2021-01-04

**Authors:** Waleed M. Sweileh

**Affiliations:** Department of Physiology, Pharmacology/Toxicology, Division of Biomedical Sciences, College of Medicine and Health Sciences, An-Najah National University, Nablus, Palestine

**Keywords:** Anti-microbial stewardship, Antimicrobial resistance, Infectious diseases, Outbreaks, Bibliometric analysis

## Abstract

**Background:**

The World Health Organization recommended the implementation of antimicrobial stewardship (AMS) in the clinical settings to minimize the development and spread of antimicrobial resistance (AMR). The current study aimed to assess global research activity on AMS as one measure for efforts dedicated to contain AMR.

**Method:**

A bibliometric method was applied using Scopus. A validated search query was implemented. Bibliometric indicators and mapping were generated. The study period was from 1990 to 2019. The search query utilized the keywords “antimicrobial stewardship” or “antibiotic stewardship” in the titles or abstracts. In addition, documents with the term “restrict” or “restriction” if used with the terms “antimicrobial” or “antibiotic” were retrieved.

**Results:**

The search query returned 4402 documents. The keyword “antimicrobial stewardship” returned 2849 documents while the keyword “antibiotic stewardship” returned 1718 documents. The terms restrict/restriction and antimicrobial/antibiotics returned 209 documents. The number of publications and cumulative citations showed a steep and parallel increase in the last decade. The region of the Americas returned the most while the Eastern Mediterranean region returned the least. The United States (*n* = 1834, 41.7%) ranked first. Main research themes in the retrieved literature were the (1) impact of AMS on hospital length stay, (2) role of pharmacists, and (3) development of resistance of various pathogens. *Clostridium difficile* (*n* = 94) and *Staphylococcus aureus* (*n* = 76) were among the most frequently encountered author keywords. The *Infection Control and Hospital Epidemiology* journal ranked first (*n* = 245, 5.6%, h-index = 134) while documents published in the *Clinical Infectious Diseases* journal (h-index = 321) received the highest number of citations per document (70.7). At the institutional level, the US *Centers for Disease Prevention and Control* (*n* = 93, 2.1%) ranked first followed by the *Imperial College London* (*n* = 86, 2.0%). The main funding sponsors were the *National Institute of Health*. Pfizer, Merck, and Bayer pharmaceutical companies played a key role in funding AMS research. International research collaboration between developed (*n* = 3693, 83.9%) and developing countries (*n* = 759, 17.2%).

**Conclusion:**

The fight against AMR is a global responsibility and implementation of AMS need to be carried out across the globe. International research collaboration between developing and developed countries should be encouraged.

## Background

Antimicrobial agents lose their activity with time because microbes, mostly on a genetic basis, develop resistance to medications [1]. Antimicrobial resistance (AMR) is accelerated upon misuse and overuse of antimicrobial agents [2]. Many reports from different parts of the world indicated a high prevalence of inappropriate or incorrect use of antibiotics both in hospitals and in primary healthcare centers [3–8]. The *Global Action Plan* (GAP) on AMR, endorsed by the World Health Organization (WHO) in 2015, considered the optimization of antimicrobial use as one of the important strategic objectives that should be included in developing national action plans to combat AMR [9]. In 2017, the *“Political Declaration of the High-Level Meeting of the General Assembly on AMR”* reaffirmed that the third goal in *Sustainable Development Goals* (SDGs) cannot be attained without tackling the problem of AMR [10]. In 2019, the WHO listed AMR as one of the top ten global health threats [11].

Antimicrobial Stewardship (AMS) has been defined as “the optimal selection, dosage, and duration of antimicrobial treatment that results in the best clinical outcome for the treatment or prevention of infection, with minimal toxicity to the patient and minimal impact on subsequent resistance.” [12]. The AMS programs have three general goals: (1) deliver the optimum antimicrobial therapy, (2) minimize misuse and abuse of antimicrobial agents, and (3) minimize the development of antimicrobial resistance [13, 14]. The AMS programs are important in hospital settings where AMR is high and poses a real threat to hospitalized patients [15–17]. In the past decade, reports on AMR in gram-negative bacteria has increased and calls for urgent action were made by international health organizations [18]. According to the United States Centers for Disease Control and Prevention (US CDC), each year in the U.S., at least 2.8 million people are infected with antibiotic-resistant bacteria or fungi, and more than 35,000 people die as a result [19]. The WHO has developed and published practical guidelines on how to optimize the use of antimicrobials by implementing AMS [20].

Research activity on Amiss an indicator of the extent of awareness of researchers and healthcare providers of the importance national and international health security given that the number of new effective antibiotics is limited and risks of serious infections is still valid. Research activity on AMS helps better future planning in the fight against AMR. Research activity, in general, reflects the commitment of governments and international health organizations in funding research related to important practical issues for the safety of human beings. The bibliometric analysis and data visualization have been widely used tools to measure and evaluate scientific research quantitatively and qualitatively [21, 22]. At least 10 bibliometric studies on AMR have been published [23–27]. However, none was published on AMS. Gaining knowledge about the published literature on AMS is of high value since it shed light on the national contribution to this field. The bibliometric analysis provides information for comparative purposes among different countries [28]. Furthermore, bibliometric data provides information about research volume and activity of different institutions for better allocation of funding. There are several scientific databases including Web of Science, Scopus, PubMed, and Google Scholar that would bring out the scientific research metrics available in the literature. Scopus database owned by Elsevier is 100% inclusive of PubMed and included twice the number of journals indexed in the Web of Science [29].

The current study aimed to use the Scopus database, which is large and provide metric analytics, to shed light on the scientific publications on AMS. The analysis focused on describing the most productive journals, institutions, authors, citations, and countries, as well as the characteristics of the relevant documents.

## Methods

### Database used

The current study used bibliometric methodology for quantitative description of the literature on AMS published in peer-reviewed journals. Grey literature such as government reports and brochures were not included in the analysis. Data used in the current study were retrieved from Scopus database since it is the largest database [29] and commonly used in the bibliometric analysis [23, 30, 31]. In bibliometric studies, usually, one database is used because bibliometric indicators and literature mapping are difficult to perform on documents retrieved from different databases. Scopus is practically 100% inclusive of PubMed and has double the number of indexed journals compared to Web of Science [29]. Therefore, Scopus is considered comprehensive and inclusive of publications present in both PubMed and Web of Science.

### Search query

The advanced search function was used in Scopus to allow for developing comprehensive search queries that include different Boolean operators. Before entering the search query, the authors did a literature review on articles about AMS to have a clear idea about all possible keywords used in AMS literature [32–39]. No previous bibliometric studies on AMS were previously published. Therefore, the search query was uniquely developed for the current study. The search query used in the current study utilized the keywords “antimicrobial stewardship” or “antibiotic stewardship (ABS)” in the titles or abstracts. This approach will retrieve the bulk of literature on AMS. However, there are certain publications in which the keywords AMS or ABS were not mentioned explicitly and therefore a second search scenario was added to the query. For example, the term “restrict” or “restriction” if used with the terms “antimicrobial” or “antibiotic” will retrieve certain documents on AMS. The full list of terms used in the second scenario included: preauthorization or pre-authorization or audit or feedback or stream-lining or streamlining or discontinuation or de-escalation or de-escalation or optimization or step-down or stepdown or education or program* or control or “quality assurance” or “decision support” or intervention or program or restrict*. The study period was from 1950 to 2019.

### Validation of the search query

In the current study, the search query was validated using three criteria. The first criteria was the judgment of two external colleagues in the field of health sciences on 100 documents sent to them as an Endnote file. The reviewers had to judge on the presence of false-positive results. The principal investigator was the final judge in case of disagreement. The absence of false-positive results was used as an indicator of validity. The author kept fine-tuning the search query until the two reviewers gave feedback on the absence of false-positive results. The second criterion of validity was the relevancy of the top 20 journals to the topic of AMS. In the final search query, the top 20 active journals were mostly in the field of infections, antimicrobials, and health. The third criterion of validly was the comparison of the research output of the top ten active authors with the number of documents on AMS for the same authors present in their Scopus profile. For example, Pulcini, C. (*n* = 57 documents), Srinivasan, A. (*n* = 42 documents), Goff, D. A (41 documents) and Newland, J. G (41 documents) while their Scopus profile indicated the following numbers 55, 41, 41, and 41 respectively. *Pearson* correlation test between the retrieved numbers and the actual numbers of ten selected authors gave a significant, positive, and strong correlation (*p* = 0.002, *r* = 0.945) suggesting a high validity of the search strategy.

### Data export and analysis

The retrieved data was exported to Microsoft Excel for tabulation. The exported data included the type of documents, annual number of publications, author names, journals, countries, institutions, funding agencies, and number of citations. Only the top ten active authors, countries, journals, institutions, and funding agencies were listed. A linear graph was created to present the annual growth of publications. Bar chart graph was created to present the extent of research collaboration for active countries. The linear and bar chart graphs were created using Statistical Package for Social Sciences (BM SPSS Statistics for Windows, Version 24.0. Armonk, NY: IBM Corp.).

### Geographic distribution of the retrieved documents

For geographic distribution of publications, the WHO classification of world regions was used: African region (AFRO), Region of the Americas (AMRO), South-East Asia Region (SEARO), European Region (EURO), the Eastern Mediterranean Region (EMRO), and Western Pacific Region (WPRO). The WHO classification was used because the WHO is in the front line for fighting AMR.

### International research collaboration

Inter-country (international) versus intra-country (local) research collaboration for the top ten active countries was assessed. Furthermore, the research collaboration between the top ten active countries and developing countries with minimum research output of 10 documents was mapped. In the current study, the International Monetary Fund was used for the classification of countries [40].

### Network visualization maps

The network visualization map of the most frequent author was created by VOSviewer program [41]. In the network visualization map, the node size is proportional to the number of occurrences while the distance between the terms measures the strength of the relation between the terms with closer distance implying a stronger relation.

## Results

The keyword “antimicrobial stewardship” returned 2849 documents while the keyword “antibiotic stewardship” returned 1718 documents. The terms restrict/restriction and antimicrobial/antibiotics returned 209 documents. The overall search query returned 4402 documents.

**Fig. 1:**
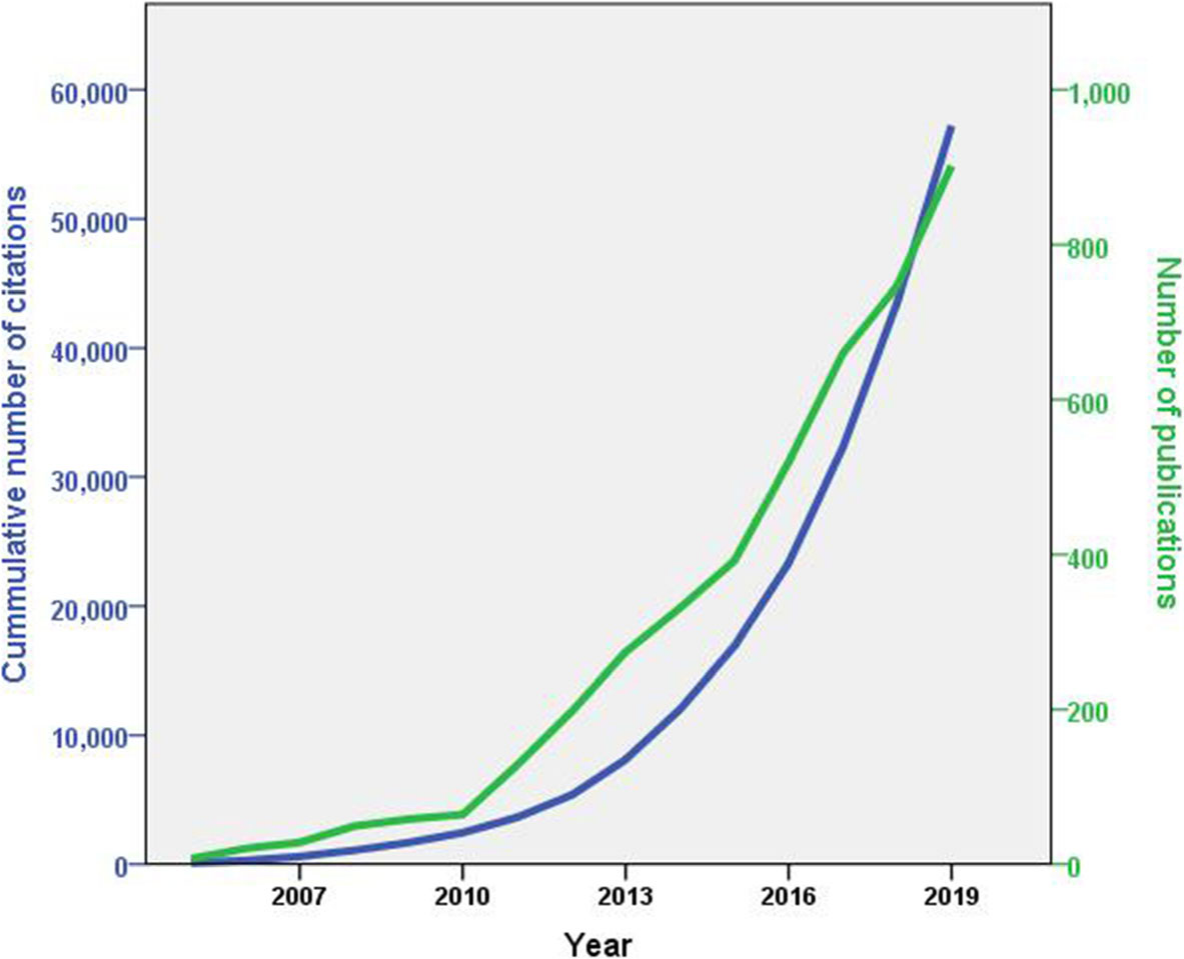
Annual growth of publications and cumulative citations on AMS

The earliest retrieved literature on AMS started in 1996 with a review article published in the *New Horizons: Science and Practice of Acute Medicine* [42]. The article discussed the relationship between antibiotic restriction (stewardship) and the development of antibiotic resistance. The growth of publications on AMS remained low from 1996 to 2010 (*n* = 252, 5.7%) followed by a steep increase from 2011 to 2019 (*n* = 4150, 94.3%). The number of publications in 2019 was 14 times higher than that in 2010. The retrieved documents have an h-index of 96. Figure 1 shows an increasing trend in the annual number of publications and the number of cumulative citations during the study period.

The retrieved documents were mainly research articles (*n* = 3065, 69.6%) and review articles (*n* = 853, 19.4%). Most of the retrieved documents were published in English (*n* = 4155, 94.4%). The remaining non-English documents were in German (*n* = 133, 3.0%), French (*n* = 43, 1.0%) or Spanish (*n* = 43, 1.0%).

**Fig. 2:**
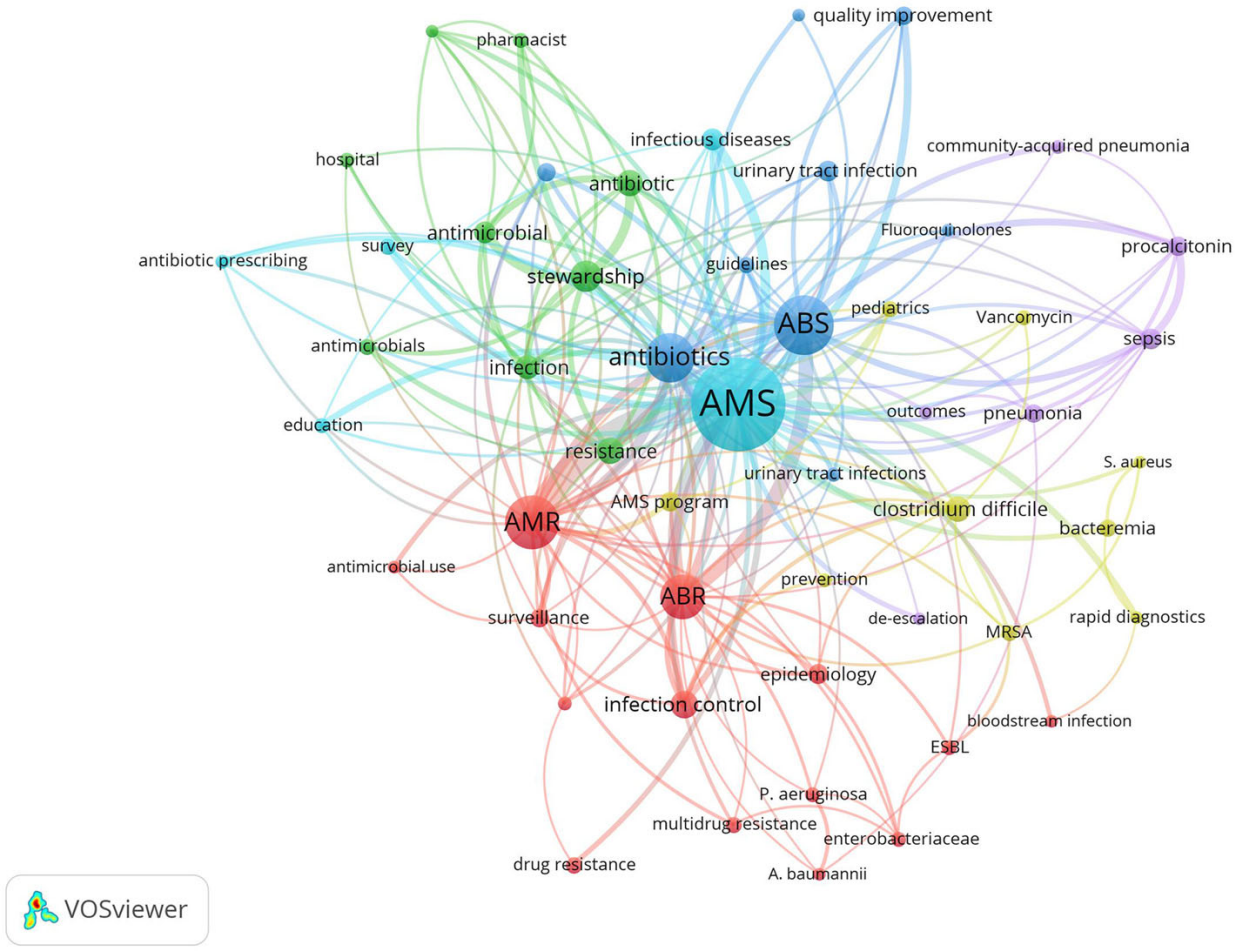
Network visualization map of most frequent author keywords

Network visualization (Fig. 2) of author keywords indicated that AMS, ABS, AMR, antibiotics, ABR, infection control, *Clostridium difficile*, urinary tract infection, procalcitonin, surveillance, pneumonia, MRSA, and pediatrics were most frequent. The terms in Fig. 2 were presented in Table 1 with their corresponding number of occurrences.

**Table 1:** Most frequent author keywords in AMS literature

Keyword	Number of occurrences	Keyword	Number of occurrences	Keyword	Number of occurrences	Keyword	Number of occurrences
antimicrobial stewardship	819	urinary tract infection	69	drug resistance	44	antibiotic prescribing	35
antibiotic stewardship	383	sepsis	67	guidelines	44	urinary tract infections	35
antimicrobial resistance	318	antimicrobial stewardship program	63	multidrug resistance	44	community-acquired pneumonia	34
antibiotics	276	epidemiology	61	survey	43	outcomes	34
antibiotic resistance	239	procalcitonin	61	education	42	rapid diagnostics	32
stewardship	129	surveillance	58	vancomycin	42	acinetobacter baumannii	31
infection control	106	quality improvement	57	esbl	40	antimicrobial use	31
resistance	101	antibiotic use	54	hospital	40	de-escalation	31
antibiotic	98	pneumonia	54	*Pseudomonas aeruginosa*	39	emergency department	30
*Clostridium difficile*	94	bacteremia	52	enterobacteriaceae	38	bloodstream infection	29
infection	84	mrsa	47	pharmacist	38	fluoroquinolones	29
antimicrobial	75	antimicrobials	46	intensive care unit	37	long-term care	29
infectious diseases	74	pediatrics	46	prevention	36	*Staphylococcus aureus*	29

*AMS* antimicrobial stewardship

**Fig. 3:**
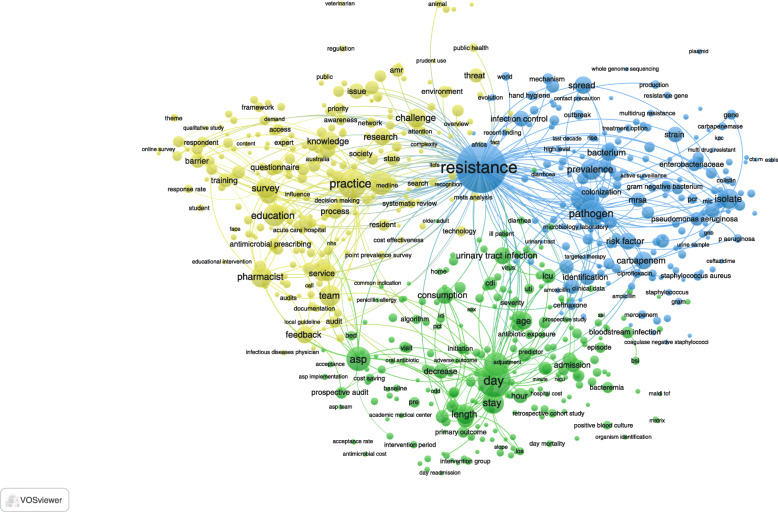
Network visualization map of most frequent terms in the abstracts of the retrieved literature

Mapping of frequent terms in the abstracts of the retrieved documents gave three major clusters representing three major research themes. The three major research themes represented 686 articles with closely related abstract terms. The first research theme (red cluster, *n* = 255 terms) discussed the knowledge, education, and practice of pharmacists about AMS. The second cluster (green cluster, *n* = 194 terms) discussed the impact of AMS programs on length of hospital stay. The third research theme (blue cluster, *n* = 237 terms) discussed the relationship between AMS programs and the development of resistance in various types of pathogens (Fig. 3).

**Fig. 4:**
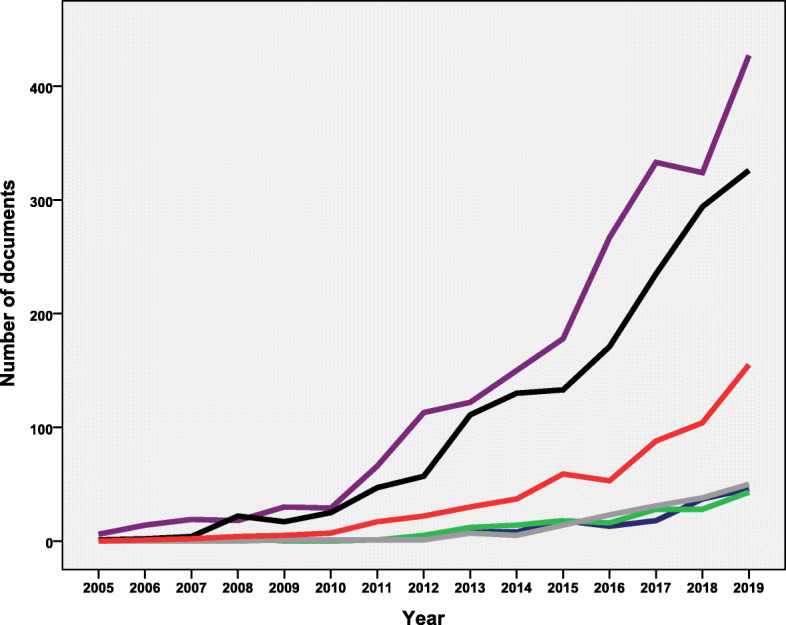
Annual growth of publications on AMS in each WHO region (Purple line: the region of the Americas. Black line: the European region. Red line: the Western Pacific region. The remaining overlapping lines represent the Eastern Mediterranean region, African region, and the South-East Asian region

Analysis of the retrieved documents based on geographical origin indicated that 172 (3.9%) documents were from AFRO, 168 (3.8%) from SEARO, 2111 (48.0%) from AMRO, 1583 (36.0%) from EURO, 157 (3.6%) from the EMRO, and 584 (13.3%) from WPRO. Publications from the AMRO region has the steepest increase in the number of publications. The EMRO, AFRO, and SEARO had similar growth pattern of publications which started after 2010 (Fig. 4). In 2019, the number of published documents for AMRO, EURO, WPRO, EMRO, SEARO, and AFRO was 427, 326, 155, 46, 43, and 50 documents respectively.

The list of top ten active countries included seven European countries, two in North America and one in the Western Pacific region. The USA led with 1834 (41.7%) documents followed distantly by the UK with 603 (13.7%) documents. Table 2 shows the list of the top ten active countries.

**Table 2:** Top ten active countries on AMS research

Rank	Country	Frequency	% ***N*** = 4402
1st	United States	1834	41.7
2nd	United Kingdom	603	13.7
3rd	Australia	284	6.5
4th	Germany	268	6.1
5th	Canada	253	5.7
6th	France	213	4.8
7th	Italy	199	4.5
8th	Netherlands	165	3.7
9th	Spain	140	3.2
10th	Switzerland	122	2.8

*AMS* antimicrobial stewardship

**Fig. 5:**
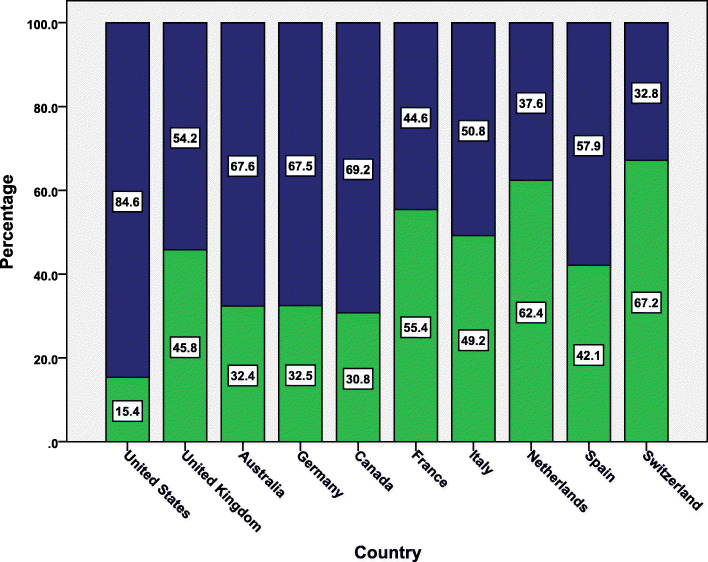
International research collaboration for top ten active countries**.** Green bars represent multiple country publications (international/inter-country collaboration) while the blue chart represent single country publication (intra-country publication)

Figure 5 presented the percentage of inter- versus intra-country research collaboration for each of the top ten active countries. The USA had the least percentage of documents with international authors (15.4%) while Switzerland had the highest percentage of documents with international authors (67.2%). The mean percentage of documents with international authors for the top ten active authors was 57%.

**Fig. 6:**
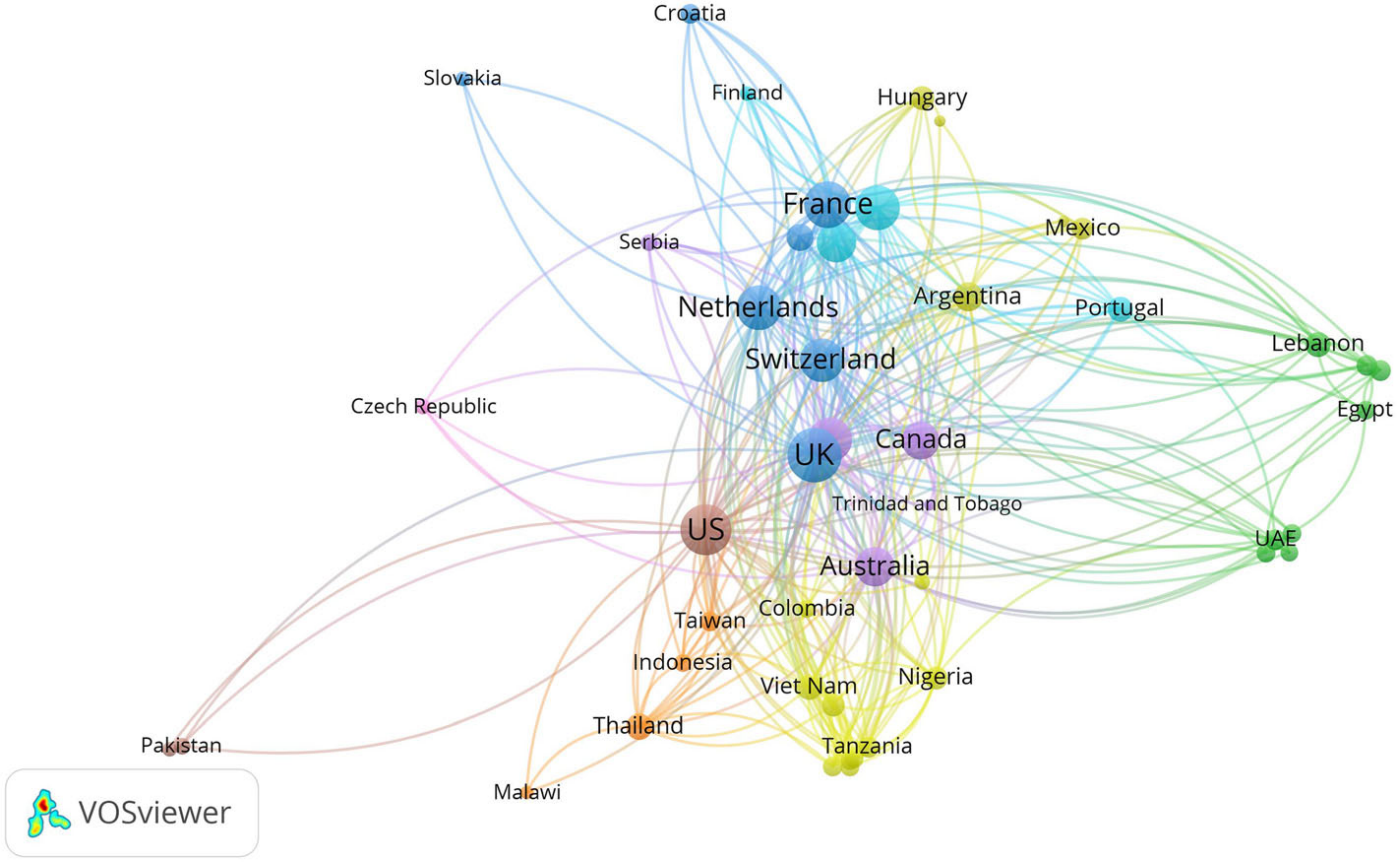
Network visualization map of research collaboration between top ten active countries (developed countries) and 37 developing countries. Each of the developing countries has a contribution of 10 documents at least

The majority of the retrieved documents (*n* = 3693, 83.9%) were published by developed countries while the remaining were published by developing countries (*n* = 759, 17.2%). The research collaboration between the top ten active countries and developing countries with a minimum contribution of 10 documents was mapped. The map included 47 countries; ten developed countries which appeared in the active list and 37 developing countries. The map showed that the active countries (developed) were in the center of the map and within a close distance to each other. The connecting lines between the developed countries were thick suggestive of relatively strong research collaboration. The connecting lines between developing and developed countries were thin suggestive of relatively weak research collaboration (Fig. 6).

Table 3 shows the top ten active institutions. The US CDC (*n* = 93, 2.1%) ranked first followed by the Imperial College London (*n* = 86, 2.0%) and the Health Protection Agency (86; 1.8%). The list of active institutions included six academic and four non-academic institutions.

**Table 3:** Top ten active institutions/organizations on AMS research

Rank^**a**^	Institution/Organization	Frequency	% ***N*** = 4402	Country
1st	*Centers for Disease Control and Prevention*	93	2.1	USA
2nd	*Imperial College London*	86	2.0	UK
3rd	*Health Protection Agency*	80	1.8	UK
4th	*University of Toronto*	79	1.8	Canada
5th	*University of Pennsylvania*	74	1.3	USA
6th	*Public Health England*	71	1.6	UK
7th	*University of Melbourne*	70	1.6	Australia
8th	*The Children’s Hospital of Philadelphia*	63	1.4	USA
9th	*Monash University*	56	1.3	Australia
9th	*The University of Utah*	56	1.3	USA

*AMS* antimicrobial stewardship

^a^In ranking, two equally active institutions/organizations were given similar ranks and one position in the rank was skipped

Table 4 shows the top ten active journals. The *Infection Control and Hospital Epidemiology* journal (*n* = 245, 5.6%) ranked first followed by the *Journal of Antimicrobial Chemotherapy* (*n* = 176, 4.0%). The majority of active journals were based in the US or the United Kingdom (UK) and all active journals ranked Q1 in the field of infectious diseases. Documents published in the *Clinical Infectious Diseases* received the highest number of citations per document (70.7).

**Table 4:** Top ten active journals on AMS research

Rank^**a**^	***Journal***	Frequency	% ***N*** = 4402	Citations per document	Country	Journal Rank
1st	*Infection Control And Hospital Epidemiology*	245	5.6	16.2	UK	Q1
2nd	*Journal Of Antimicrobial Chemotherapy*	176	4.0	24.5	USA	Q1
3rd	*Clinical Infectious Diseases*	134	3.0	70.7	UK	Q1
4th	*American Journal Of Infection Control*	123	2.8	12.6	USA	Q1
5th	*Plos One*	75	1.7	15.5	USA	Q1
6th	*International Journal Of Antimicrobial Agents*	68	1.5	17.7	Netherlands	Q1
7th	*American Journal Of Health System Pharmacy*	67	1.5	13.7	USA	Q1
7th	*Antimicrobial Resistance And Infection Control*	67	1.5	10.4	UK	Q1
9th	*Journal Of Hospital Infection*	63	1.4	12.5	UK	Q1
10th	*Clinical Microbiology And Infection*	61	1.4	26.2	UK	Q1
10th	*Open Forum Infectious Diseases*	61	1.4	5.5	USA	Q1

*AMS* antimicrobial stewardship

^a^In ranking, two equally active journals were given similar ranks and one position in the rank was skipped

Q1 = first quartile = highest rank. The information regarding journal ranking was obtained from Scimajo Journal Rank

**Fig. 7:**
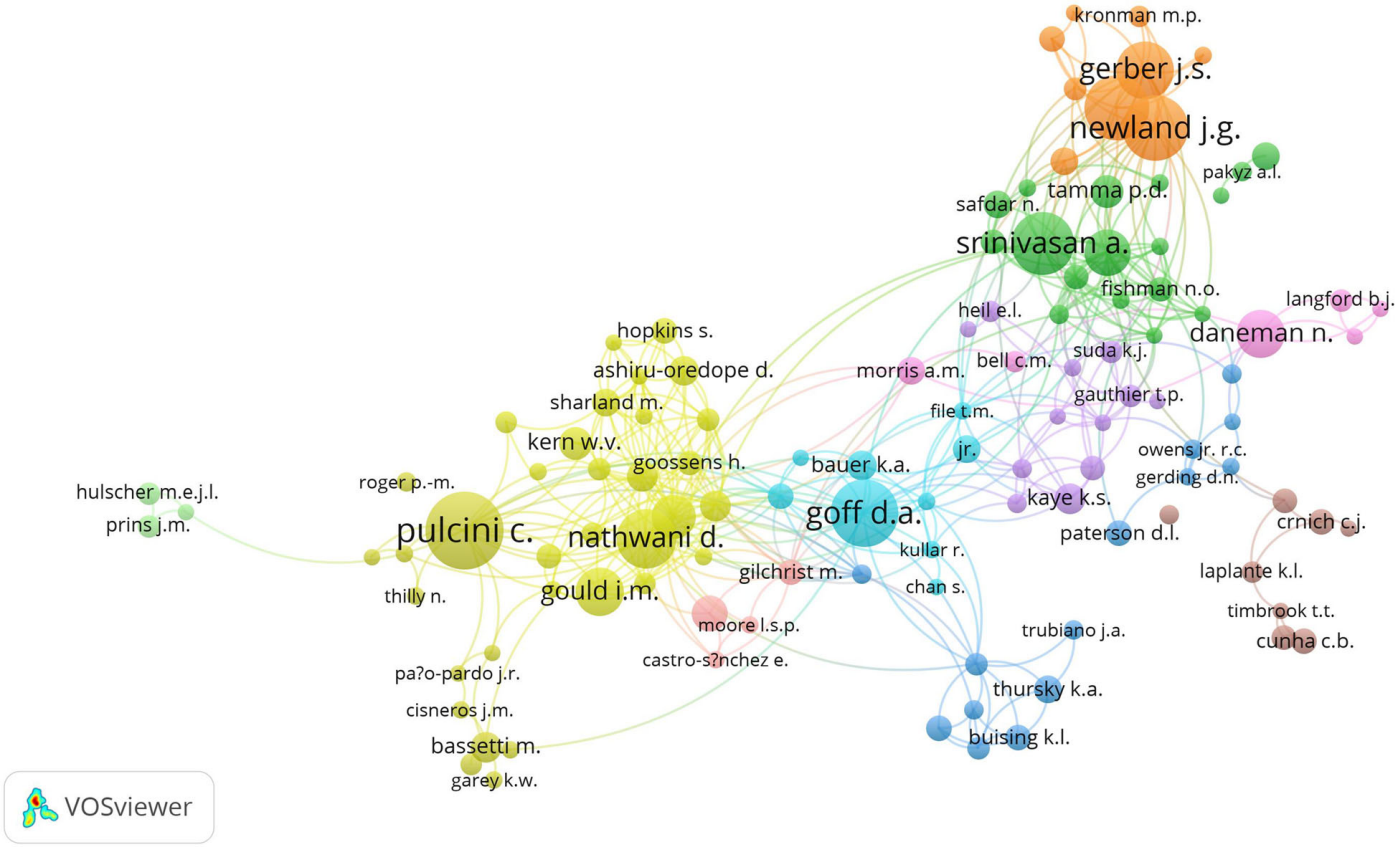
Network visualization map of authors with minimum contribution of 10 documents The map included 116 authors. There were nine authors who did not fit into any research group and were not shown in the map

The total number of authors publishing on AMS was 15,225, of which 125 (0.8%) published more than ten documents and 14,462 (96.3%) authors published less than five documents. The co-authorship network map of authors with a minimum of 10 publications is shown in Fig. 7. Authors with the largest node size contributed the most and included Pulcini, C.; Srinivasan, A.; Goff, D.A.; Newland, J.G.; Hersh, A.L.; Nathwani, D.; Gerber, J.S.; Gould, I.M.; Daneman, N.; and Cosgrove, S.E.

Analysis of funding showed that 1928 (43.8%) documents on AMS were funded. The *National Institute of Health* (*n* = 158, 3.6%) was the most active funding sponsor followed by the international pharmaceutical Companies; Pfizer (*n* = 68; 1.5%) and Merck (*n* = 56, 1.3%). Other pharmaceutical companies such as Bayer (*n* = 20, 0.5%) and AstraZeneca (*n* = 19, 0.4%) were among the top active funding sponsors.

## Discussion

In the current study, peer-reviewed literature on AMS was investigated and descriptive indicators were presented. The current study showed a 14-fold increase in the number of publications in the last decade. This increase came as a result of (1) the seriousness and the global dimension of AMR [43], (2) the important role of the 574 implemented AMS programs in improving certain patient health outcomes and cost reduction [39, 44], (3) the increasing calls and recommendations made by health organizations such as the SHEA and *Infectious Diseases Society of America* (IDSA) to combat AMR [45–47], and (4) the awareness of WHO member states to implement policies to achieve objective number 4 of the GAP to combat AMR [9].

The current study indicated that authors from the US have the highest contribution to the retrieved documents. In 2014, the US CDC called on all hospitals in the USA to implement antibiotic stewardship programs and released the guidelines for implementing such stewardship (*Core Elements of Hospital Antibiotic Stewardship Programs*) [48]. In 2015, “*The United States National Action Plan for Combating Antibiotic Resistant Bacteria*” set a goal for implementation of the Core Elements in all hospitals that receive federal funding [49]. This means that the control of AMR requires active involvement of the policy changes. The WHO released a toolkit in 2019 to implement AMS in low- and middle-income countries [9] to facilitate and help low- and middle-income countries in combating AMR. It should be emphasized that the steep growth in the number of publications does not necessarily imply the effectiveness of AMS in reducing antimicrobial resistance. A recent systematic review on the quality of studies evaluating antimicrobial stewardship interventions concluded that the overall quality of antimicrobial stewardship studies is low with no clinical and microbiological outcome data [37]. Another recent systematic review on the effectiveness of AMS concluded that there is no solid evidence that AMS programs are effective in reducing antibiotic resistance in hospital settings and further stronger studies are needed to reach solid conclusions [34].

The current study indicated that *C. difficile* was frequently encountered author keyword in the retrieved literature on AMS [50–58]. In the 2019 Antibiotic Resistance Threats Report, the U.S. CDC has declared *C. difficile* infections as an urgent threat [59, 60]. In the European point prevalence study, *C. difficile* ranked sixth among microorganisms responsible for healthcare-associated infections [61]. It is believed that the majority of *C. difficile* in the United States are hospital-acquired [62]. A recent systematic review of 229 publications from 41 countries that the overall rate of CDI in healthcare facilities was 2.24 (95% confidence interval CI = 1.66–3.03) per 1000 admissions/year [63]. The impact of AMS programs on CDI is controversial. Certain studies indicated that AMS caused a significant reduction in *C. difficile* infections [52, 53] while other studies did not [50, 57, 58].

In the current study, rapid diagnosis of causative microbes was among the most frequent author keywords found in AMS literature. Effective antimicrobial stewardship is closely linked with the availability of techniques that can make correct and rapid diagnoses. For example, many types of viral respiratory infections are clinically indistinguishable from bacterial respiratory infections which leads to overuse or misuse of broad-spectrum antibiotics [64, 65]. Rapid diagnostic methods are important for implementing effective AMS programs given that traditional microbiological testing methods requires 2–4 days resulting in empirical treatment with strong and broad- spectrum antibiotics before the results of microbiological diagnostics are known [66]. Therefore, rapid diagnostics is a key goal of AMS to reduce unnecessary antibiotic use [67]. Integration of rapid diagnostic testing in AMS programs has the potential for early organism identification with significant improvement in patient outcomes and cost [68–73]. Rapid diagnostic tools and microbial identification along with the appropriate antibiotic administration is critical in patients with serious infections such as sepsis [74–78]. Therefore, rapid diagnostic and identification tools provide an excellent opportunity for all healthcare specialists to collaborate and reach a rational and timely decision in critical situations. The implementation of new technology for rapid diagnosis in AMS programs might increase the cost and reduce savings [79, 80]. Studies indicated that evidence for the advantages of rapid tests in bloodstream infections seems strong while that for respiratory and gastrointestinal infections are still poor [74, 81–83].

The current study showed that the bulk of the publications on AMS came from the AMRO and EURO. The leading role of these two regions in AMS was also visible in other health-related fields [84, 85]. The presence of the US CDC and European CDC and various specialized academic and governmental institutions in public health and infectious diseases gave the region of the Americas and the European region a leading role.

The current study showed that the contribution of China to the literature on AMS was not visible in the top active list. China participated in publishing 99 documents (data not shown) while Switzerland which ranked 10th in the list participated in publishing 122 documents. Most of the published document from China originated from academic institutions and the Chinese CDC did not play a key role in these publications. It is possible that most publications on stewardship from China were published in national Chinese journals or as grey literature, i.e. reports, governmental brochures, articles in newspapers and others. In the current study, such grey literature was not included. The same argument applies to other world regions and countries which showed a limited contribution to this subject. Antimicrobial consumption increased by 79% in China between 2000 and 2015, which was higher than the increase in global antimicrobial consumption [86]. The Chinese government took multiple measures to strengthen the AMS to improve the intelligent use of antibiotics and therefore to combat the increasing rates of AMR in China [87, 88]. In 2016, China also launched its National Action Plan to Contain Antimicrobial Resistance (2016–2020) mainly to optimize antimicrobial consumption and antimicrobial resistance [89]. It should be emphasized that active journals in publishing documents on AMS were based in the USA and Europe. This might have created a certain bias toward countries in which these prestigious journals and publishers are based.

International research collaboration is known to increase research productivity and impact [90–92]. It is expected that the global situation of AMR is worsened by limited international research collaboration. Most policymakers in different world regions are keen to implement the WHO recommendation of implementing AMS. However, the limited number of experts and specialized institutions might hinder researchers in many countries to investigate AMS. International research collaboration is important for countries in AFRO, EMRO, Latin America, and SEARO where experts can help in assessing the situation and help implement AMS along with other strategies. The current study showed limited research collaboration in the field of AMS between developed and developing countries. Several studies discussed international research collaboration in different fields [93, 94]. A relatively recent study on AMR in the Asia-Pacific region recommended inter-country collaboration to contain the escalating rates of AMR [95]. A second recently published article with the title “Challenges and opportunities for antimicrobial stewardship in resource-rich and resource-limited countries” discussed in details the challenges in implementing AMS programs in rich and poor countries [96]. Collaboration in the field of AMS and AMR research between developed and developing countries is extremely important for global health security given the limited knowledge and experience in most developing countries about AMR [97–103].

The current study also showed that publications on AMS had a high scientific impact as assessed by the *h*-index relative to other publications in the field of microbiology, infections, and antimicrobials [23, 25, 26, 104–106]. This indicates that the subject is of interest to a diverse number of readers and researchers. The association between AMS and AMR is one potential reason for attracting many citations. The annual campaigns by the WHO to increase awareness of the public about antibiotic resistance was also a key element in drawing attention to this subject [107]. Many several other agencies played an influential role in promoting AMS research and implementation such as Alliance for the Prudent Use of Antimicrobials, British Society for Antimicrobial Chemotherapy, US CDC, European Centre for Disease Prevention and Control, HealthCare Infection Control Special Interest Group, IDSA, SHEA, and The Dutch Working Party on Antibiotic Policy. Several campaigns were carried out by these agencies to improve antimicrobial use such as “Get Smart” from the US CDC and European Antibiotic Awareness Day. Endorsement of the G20 to take action against AMR gave momentum to research activity and citations in this field [108]. Publishing more than a quarter of the retrieved documents in leading journals in the field of infectious diseases and antimicrobial therapy also played a positive role in attracting a larger number of citations [109–111]. Finally, the heavy involvement of governmental and non-governmental bodies, as well as multinational huge pharmaceutical companies in funding research on AMS, attracted the attention of clinicians, scientists, and healthcare providers to the significance of this subject. All these factors are known to increase research activity and citations to a certain subject [109–112].

### Limitation

The current study has a few limitations. The literature on AMS has been retrieved from journals indexed in Scopus while grey literature and publications in non-indexed journals have not been studied. This means that a certain number of publications particularly from EMRO, SEARO, and AFRO where most national journals are not indexed in Scopus has been missed. If this is the case, then the current study has underestimated the productivity from these regions. Another limitation is the method of counting publications for countries, authors, and institutions. Scopus counts a document once for each author. The same applies for counting documents for countries and institutions when there were different affiliations on the same document. This means that there was an overlap in the results and that the results of certain countries, institutions, and active authors might be over-estimated. Finally, the search query was developed to retrieve all potential documents in the field of AMS. However, the presence of false-positive or false-negative results remains a possibility.

## Conclusions

The current study is the first bibliometric study on AMS. The study came as a response to global calls to strengthen the fight and increase awareness about AMR. The current study showed skewed results toward high-income countries. The focus of the current literature on AMS was directed toward several themes such as reduction of antimicrobial use, cost-effectiveness, pharmacist knowledge and practice, length of hospital stay, rapid diagnostics of microbes in critical settings, and impact on microbial resistance. Research and implementation of AMS need to be globalized and given a priority in the fight against AMR. National action plans in low and middle-income countries need to provide funding for implementation, research, and awareness campaigns about AMR to fulfill the commitment of containment of AMR. Researchers and clinicians in low- and middle-income countries need to establish connections and collaborations with peers in high-income countries to implement and carry out research on AMS. The global effort needs to be coordinated to fight AMR and fulfill the international goals of SDGs.

## Data Availability

All data presented in this manuscript are available on Scopus database using the search query listed in the methodology section.

## Abbreviations

AMS: Antimicrobial stewardship
AMR: Antimicrobial resistance
GAP: Global Action Plan
WHO: World Health Organization
CDC: Centers for Disease Control and Prevention
USA: United States of America
UK: United Kingdom
Q1: First Quartile
SDG: Sustainable Development Goal
ECDC: European Centers of Disease Control and Prevention
